# Supplementary material for: Content-rich biological network constructed by mining PubMed abstracts
Source: BMC Bioinformatics. 2004 Oct 8;5:147. doi: 10.1186/1471-2105-5-147 (PMC528731; doi:10.1186/1471-2105-5-147)
Supplement: Additional File 2 — The original results of the above study (non-essential files are deleted to keep the file size under the limit set by BMC bioinformatics). [file 1471-2105-5-147-S2.bz2 › chilibotAdditionalFile2/dip50/477ID10734104E10562/html/CCL2_CCRL1.html]

 


 **CCL2** and **CCRL1** 
  
Found 120 abstracts in PubMed, retrieved 50.  
 

 What does Google say? 
 PDF only 
| .edu only 

---

**Interactive relationship** (e.g. stimulation, inhibition, etc)

**Neutral relationship**- RA synovial tissue RANTES regulated upon activation, normally T cell expressed and secreted chemokine and monocyte chemoattractant protein 1  [ **CCL2** ]  immunoreactive cells colocalized with CCR1  [ **CCRL1** ]  and CCR2, respectively, on serial sections.  Ref: 11352233 Arthritis Rheum, 2001
- Inhibition of labeled MIP 1alpha binding was achieved with unlabeled MIP 1alpha and RANTES, the latter a beta chemokine that binds to both CCR1  [ **CCRL1** ]  and CCR5, but not by MCP 1  [ **CCL2** ] .  Ref: 11032879 J Neurochem, 2000
- Labeled MIP 1alpha binding was also antagonized by unlabeled MCP  [ **CCL2** ]  3, which is also recognized by CCR1  [ **CCRL1** ] , and MIP 1beta, which is a ligand for CCR5.  Ref: 11032879 J Neurochem, 2000
- Therefore, it is concluded that glomerular MCP 1  [ **CCL2** ]  and RANTES production directs circulating leukocytes that express CCR1  [ **CCRL1** ] , CCR2, and CCR5 into the glomerulus.  Ref: 11316850 J Am Soc Nephrol, 2001
- Lastly, we present evidence that fresh patient tumor cells express mRNA for the MCP 1  [ **CCL2** ]  receptor, CCR2, as do myeloma cell lines along with a second MCP 1  [ **CCL2** ]  receptor, CCR11  [ **CCRL1** ] .  Ref: 12357369 Leukemia, 2002
- The present findings suggest that MCP 1  [ **CCL2** ] , RANTES, and IP 10 may participate in the pathogenesis of mercuric chloride induced tubulointerstitial fibrosis with mononuclear cell infiltration, via CCR2, CCR1  [ **CCRL1** ]  or CCR5, and CXCR3, respectively.  Ref: 12834626 Exp Mol Pathol, 2003

**Non-interactive relationship** (e.g. studied together, co-existance, homology, etc.)

- For examples, Langerhans cells migrate selectively to MIP 3 alpha via CCR6, blood CD11c DC to MCP  [ **CCL2** ]  chemokines via CCR2, monocytes derived DC respond to MIP 1 alpha beta via CCR1  [ **CCRL1** ]  and CCR5, while blood CD11c DC precursors do not respond to any of these chemokines.  Ref: 11155441 Springer Semin Immunopathol, 2000
- Only CpG ODN exacerbated HAF GN with an increase of glomerular macrophages, which was associated with massive albuminuria and increased renal MCP 1  [ **CCL2** ]   **CCL2** , RANTES CCL5, CCR1  [ **CCRL1** ] , CCR2, and CCR5 mRNA expression.  Ref: 12538732 J Am Soc Nephrol, 2003
- Chemokine inducible transcripts could be clustered in functional groups, including selected cytokines and receptors e.g., IL 1beta,  **CCL2**  monocyte chemotactic protein 1  [ **CCL2** ] , and the CCL5 receptor CCR1  [ **CCRL1** ]  and molecules involved in extracellular matrix recognition and digestion e.g., CD44 splice transcripts, urokinase type plasminogen activator receptor, matrix metalloprotease MMP 9, and MMP 19.  Ref: 11907119 J Immunol, 2002
- The most active chemokines for resting Th IL 10, in terms of calcium mobilization and in vitro migration, were in order of potency  **CCL2**  monocyte chemoattractant protein 1  [ **CCL2** ] , CCR2 ligand, CCL4 macrophage inflammatory protein 1beta, CCR5 ligand, CCL3 macrophage inflammatory protein 1alpha, CCR1  [ **CCRL1** ]  5 ligand, CCL17 thymus and activation regulated chemokine, CCR4 ligand, CCL1 I 309, CCR8 ligand, CXCL12 stromal derived factor 1, CXCR4, and CCL11 eotaxin, CCR3 ligand.  Ref: 11145678 J Immunol, 2001
- The mRNAs for MIP 1alpha and MCP 1  [ **CCL2** ] , as well as their cognate receptors CCR1  [ **CCRL1** ]  and CCR2, respectively, were localized in resident and infiltrating macrophages in numbers commensurate with those of F4 80 immunopositive macrophages in adjacent tissue sections.  Ref: 12444600 J Neurosci Res, 2002
- Whereas freshly harvested monocytes were strongly attracted by monocyte chemotactic protein 1  [ **CCL2** ]  MCP 1  [ **CCL2** ] , a specific ligand for CCR2, only a weak response was observed to macrophage inflammatory protein 1alpha MIP 1alpha, which binds to CCR1  [ **CCRL1** ]  and CCR5.  Ref: 11272275 J Leukoc Biol, 2001
- Although certain transcripts were stimulated by both agonists e.g., IL 1beta and  **CCL2** , others were induced only by either LPS e.g., TNF alpha and IL 6 or CCL5 e.g., MMP 19 or were divergently regulated e.g., CCR1  [ **CCRL1** ] .  Ref: 11907119 J Immunol, 2002
- In J774 cells, CpG ODN induced the chemokines MCP 1  [ **CCL2** ]   **CCL2**  and RANTES CCL5 and the chemokine receptors CCR1  [ **CCRL1** ]  and CCR5.  Ref: 12538732 J Am Soc Nephrol, 2003
- We used radioligand binding and 35S GTPgammaS exchange assays in membranes from a cell line transfected to express CCR1  [ **CCRL1** ]  Ba F3 hCCR1 to characterize a panel of chemokines HCC 1, MIP 1alpha, MIP 1beta, MIP 1delta, MPIF 1, MCP  [ **CCL2** ]  2, MCP  [ **CCL2** ]  3, and RANTES as CCR1  [ **CCRL1** ]  ligands.  Ref: 12381680 Br J Pharmacol, 2002
- CD14 DCs retain unique features of tissue DCs, as evidenced by the gene expression profile of no CCR7 but more CCR1  [ **CCRL1** ]  and no TARC but abundant MCP1  [ **CCL2** ]  and Eta 1.  Ref: 12176896 Blood, 2002
- We extend these findings in this report to reveal that such sites exemplify characteristics of the recognized major receptors for MCP 1  [ **CCL2** ]  and MIP 1alpha CCR2, and CCR1  [ **CCRL1** ]  and CCR5, respectively.  Ref: 11032879 J Neurochem, 2000
- The mRNA expression of CC chemokines predominantly monocyte chemoattractant protein 1  [ **CCL2** ]  MCP 1  [ **CCL2** ]   **CCL2** , RANTES CCL5 and their receptors CCR1  [ **CCRL1** ] , CCR2, CCR5 increased progressively from days 2 to 10.  Ref: 11373340 J Am Soc Nephrol, 2001
- In EAE protected mice, 17 beta estradiol strongly inhibited mRNA expression of the chemokines RANTES, MIP 1 alpha, MIP 2, IP 10, and MCP 1  [ **CCL2** ] , and of the chemokine receptors CCR1  [ **CCRL1** ] , CCR2 and CCR5 at both time points.  Ref: 11550221 J Neurosci Res, 2001
- At 26 weeks postirradiation, messages encoding the chemokines BLC now known as Scyb13, C10 now known as Scya6, IP 10 now known as Scyb10, MCP 1  [ **CCL2** ]  now known as Scya2  [ **CCL2** ] , MCP  [ **CCL2** ]  3 now known as Scya7, MIP 1gamma now known as Scya9, and RANTES now known as Scya5 and the chemokine receptors Ccr1  [ **CCRL1** ] , Ccr2, Ccr5 and Ccr6 were elevated in fibrosis sensitive C57BL 6 mice.  Ref: 11839087 Radiat Res, 2002
- In parallel, CCR1  [ **CCRL1** ]  and CCR5 expression is down regulated and CXCL8,  **CCL2** , CCL3 and CCL5 production is up regulated.  Ref: 12548563 Eur J Immunol, 2003
- In summary, in a model of transient IC GN, MCP 1  [ **CCL2** ]  and RANTES and their receptors CCR1  [ **CCRL1** ] , CCR2, and CCR5 are expressed early and are already downregulated at the peak of proteinuria and leukocyte infiltration.  Ref: 11316850 J Am Soc Nephrol, 2001
